# Combining Structural Optimization and Process Assurance in Implicit Modelling for Casting Parts

**DOI:** 10.3390/ma14133715

**Published:** 2021-07-02

**Authors:** Tobias Rosnitschek, Maximilian Erber, Christoph Hartmann, Wolfram Volk, Frank Rieg, Stephan Tremmel

**Affiliations:** 1 Engineering Design and CAD, University of Bayreuth, Universitaetsstr. 30, 95447 Bayreuth, Germany; frank.rieg@uni-bayreuth.de (F.R.); stephan.tremmel@uni-bayreuth.de (S.T.); 2 Metal Forming and Casting, Technical University of Munich, Walther-Meißner-Straße 4, 85748 Garching, Germany; maximilian.erber@utg.de (M.E.); christoph.hartmann@utg.de (C.H.); wolfram.volk@utg.de (W.V.)

**Keywords:** die casting, implicit modeling, process assurance, structural optimization, virtual product development

## Abstract

The structural optimization of manufacturable casting parts is still a challenging and time-consuming task. Today, topology optimization is followed by a manual reconstruction of the design proposal and a process assurance simulation to endorse the design proposal. Consequently, this process is iteratively repeated until it reaches a satisfying compromise. This article shows a method to combine structural optimization and process assurance results to generate automatically structure- and process-optimized die casting parts using implicit geometry modeling. Therefore, evaluation criteria are developed to evaluate the current design proposal and qualitatively measure the improvement of manufacturability between two iterations. For testing the proposed method, we use a cantilever beam as an example of proof. The combined iterative method is compared to manual designed parts and a direct optimization approach and evaluated for mechanical performance and manufacturability. The combination of topology optimization (TO) and process assurance (PA) results is automated and shows a significant enhancement to the manual reconstruction of the design proposals. Further, the improvement of manufacturability is better or equivalent to previous work in the field while using less computational effort, which emphasizes the need for suitable metamodels to significantly reduce the effort for process assurance and enable much shorter iteration times.

## 1. Introduction

Today’s product development is characterized by short times to market and the need for error-minimized product designs. Therefore, parts should be structurally optimized and process optimized since an optimized process design reduces manufacturing costs and scrap-rate [1]. These optimization tasks are time-consuming, demand a high extension of expert knowledge, and combine their results with multiple manual work steps. Consequently, there is a vast potential for digital engineering in combining structural optimization of casting parts and process assurance [1,2,3,4].

Basically, the optimization for structure and process is shared between two specialized departments, which iteratively transfer the part between each other. Each division has to prepare the obtained file for their process, and afterwards, has to redesign a new part based on the optimization results manually. This process is repeated until it reaches a satisfying compromise [1,2]. Therefore, this article addresses the combination of structural optimization and process assurance (PA) with implicit modeling to create structure- and process-optimized parts automatically.

### 1.1. Structural Optimization of Casting Parts

A common computer-aided-engineering (CAE) tool for structural optimization is topology optimization (TO), which determines the optimal distribution of the material in a given design space at predefined loads and constraints [5,6,7]. A disadvantage of TO is that the derived design proposals cannot be manufactured by casting processes and, therefore, have to be redesigned manually [8,9]. This consequently led to the development of manufacturing constraints in TO [6,7].

For instance, [10] uses a unified projection-based approach where a domain of design variables is considered and projected into a pseudo-density domain to obtain the optimized solution. This approach was used to implement manufacturing constraints like minimum feature size, symmetry, and extrusion, which can be used for casting, forging, or turning. On a high level, one can say that the manufacturing constraints limit the range of solutions to a TO problem. The simplification of the design proposal is also desired in [11] and applied by using an explicit Heaviside parametrization in gradient driven TO to limit the complexity of the solution to apply the design proposals for milling or casting processes. In [12], a fictitious physical model is proposed to consider partition lines and to avoid undercuts and voids in the design. The use of partitioned molds is also considered in [13,14,15]. These works use the partition line to imply the casting manufacturing constraints to avoid undercuts and voids. All these constraints have been derived from experience knowledge existing for the design of casting parts, knowing that these features are beneficial in increasing manufacturability in general.

Nevertheless, these approaches represent basically a simplification of the unconstraint design proposal based on production restrictions, and thus, they cannot describe the limits of stable production processes.

### 1.2. Process Simulation

In this work, two casting processes are investigated, which are applied in a wide range of engineering industries. The low pressure die casting (LPDC) process as well as the high pressure die casting (HPDC) process are both techniques in which a cavity formed by a steel mold is filled with liquid metal. Due to different casting pressures, and therefore filling velocities, these processes highly differentiate in their challenges to produce high-quality castings.

In the HPDC process, the metal is forced into the cavity by a piston with ingate velocities of 30 m/s to 60 m/s, resulting in a highly turbulent filling. Air entrapment is unavoidable, but can be kept to a minimum by choosing a good design of the part geometry and the casting system [16].

In the LPDC process, the metal is forced into the cavity with a reduced velocity in the raiser tube. The limiting velocity depends on material and geometry parameters and ensures a controlled filling and avoids air entrainment. For aluminum alloys, the critical velocity ranges from 0.3 m/s to 0.5 m/s for a channel thickness between 3 mm and 25 mm [17].

Numerical simulations of manufacturing processes can minimize the trial-and-error effort, which traditionally goes along with the development of new products and saves time and costs [18]. In the context of casting processes, numerical process simulations can be used, for instance, to analyze the fluid flow, heat transfer, solidification, or defect behavior [19]. Two of the most common numerical methods for process simulation are the Finite-Element Method, which is, for instance, used in [20] to optimize the gate system in HPDC, and the Finite-Volume Method (FVM), which is commonly used for computational fluid dynamics (CFD) [21].

Casting process simulations offer a good way to understand the defect formation in castings and ensure a stable casting process. Because of the wide range of casting processes, the evaluation of the simulation has to be done based on different simulation results. Most commercially available casting simulations use the eulerian approach of FVM for evaluating the metal flow during the filling process. The free surface between metal and air is represented by the VOF (volume of fluid) method [22]. These simulations are time consuming and computationally expensive. Other approaches, for example [23] used the lagrangian simulation technique Smoothed Particle Hydrodynamics (SPH) for the simulation of HPDC, but could not reduce the calculation time in a satisfactory way. Therefore, the use of casting simulations in computational optimization procedures with multiple iteration steps leads to high computation costs, and optimization algorithms have only been used for varying a small number of parameters [24,25,26]. Consequently, simplified models are desired to reduce the calculation time and to allow further autonomous optimizations.

### 1.3. Combining Structural Optimization and Process Assurance

The aim of this work is the integration of process knowledge into the TO of cast components. In the literature, the approaches can be found which deal with process simulation of the design and optimization of the casting system, e.g., the mold or the ingate system of a known component’s geometry. Since the mold design has a significant impact on the quality of casting processes, process simulation is often used to optimize the ingate system or mold design to optimize, respectively, the process or the quality of the casted part. For example, [27] uses process simulation to optimize the effect of cooling system location on the stresses produced in the mold, to enhance the mold life. Additionally, [28] uses CFD simulations to analyze the filling of the cavity and quantifies porosities. Based on these results, the molten metal flow systems are modified, which leads to improvements of the process and deepened knowledge about the phenomena of flow in casting processes. An approach for multi-objective optimization of the solidification of casting processes is shown in [29] by using machine learning combined with process simulations.

Nevertheless, in our approach, the component’s geometry is not known in advance, but is generated from an initial design space in an iterative process based on topology optimization and process simulations performed in parallel. Up to now, process knowledge is already taken into account in commercial TO by manufacturing restrictions [30]. A further approach shown in [3] uses the results of casting simulations to improve the manufacturability of topology-optimized components. Here, flow-induced defect sources with high vorticity and regions that are late filled as well as defect locations that cannot be fed due to too early solidification of the melt are considered [4]. The work in [2] proposes the use of Dijkstra’s shortest-path [31] algorithm for the investigation of HPDC parts. In a further work presented in [1], these results are combined with a casting simulation to filter poorly manufacturable component areas in a TO step. Another research project deals with the optimization of a parametric component using structural and process simulation [32].

To summarize, the focus from a process point of view in the literature is on optimizing the mold design and ingate system to improve the process quality for a known geometry. From a structural point, the development of manufacturing constraints was of high interest in recent years. Thus, the literature on incorporating process knowledge into the TO process on a deeper level than manufacturing constraints is scarce. The works in [1,2,3,4] incorporate full-scale casting process simulations into TO, accompanied by high iteration times, since it is expensive to obtain results for parameters like vorticity.

The gap in the literature we want to address with our research is to develop approaches to using geometry-linked evaluation criteria for HPDC and LPDC, which are potentially suited to omitting process simulation during the TO but still show sufficient information to describe the manufacturability of design proposals qualitatively. We investigate further how the evaluation criteria can be used to modify the design proposal’s geometry. Additionally, a high degree of automation is aimed at so that the whole process shifts towards being fully autonomous. Thereby, this article focuses solely on modifying the part geometry; a modification of the ingate system, as, for instance, shown in [33], is not within the scope of this article.

Hence, in this article, we present a new workflow for automatically combining TO and PA results. Based on a casting process-based criterion, the structurally optimized geometry is then modified via implicit modeling to create a design proposal that shows increased manufacturability and is, therefore, structure- and process-optimized. The distant goal is to replace the casting process simulation with a metamodel that evaluates the developed criterion. The development of this metamodel is the objective of our future work, for which the developed and proven functionality of a geometry-linked evaluation criterion is essential.

## 2. Materials and Methods

**Figure 1:**
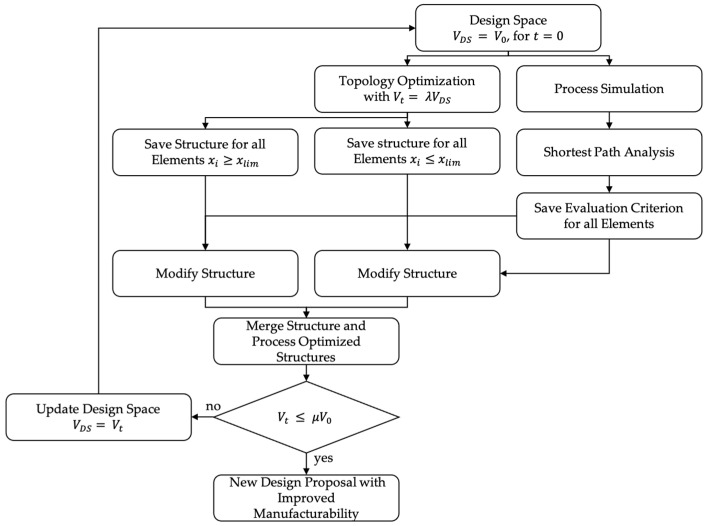
Proposed workflow of the optimization for the combination of topology optimization and process assurance. Starting from the initial design space, a TO with a volume target volume $V_{t}$ and a parallel process simulation. The One-Step Optimization ends after the first iteration. In the iterative optimization, the design space $V_{DS}$ is modified for every new iteration with a step-length of λ on the basis of previous results.

The basic idea of the workflow presented in this article is to parallel conduct TO and PA and then use implicit modeling techniques to combine the best elements of TO with the best elements of PA. Thereby, best in this context means structural optimized and process optimized, respectively. For PA, we use a CFD-based process simulation followed by the shortest path analysis and the calculation of the evaluation criterion. As depicted in Figure 1, the result of this workflow is a new design proposal that shows increased manufacturability compared to the standard TO part.

This article distinguishes between a One-Step optimization and an iterative optimization approach with a parameter λ that controls the step-length in the optimization scheme. In iterative optimization, the design space is modified for every new iteration based on previous optimization results. Therefore, the target volume of the volume constraint is given by

(1) $$V_{t}=\lambda V_{DS},$$

with step-length λ, target volume $V_{t}$, and volume of the current design space $V_{DS}$. The iterative optimization ends when

(2) $$V_{t}\leq \;\mu V_{0}$$

with μ being the globally defined volume constraint and $V_{0}$ the volume of the initial design space. Therefore, the One-Step Optimization can be seen as a special case of the Iterative Optimization with λ=μ.

In the first step, TO and process simulation are executed in parallel. From the TO results, two structures are built, one with all elements whose design variable $x_{i}$ is greater or equal than the threshold $x_{lim}$, representing the structural optimal elements; and one with all elements less than the threshold representing the structural scrap-rate.

The process simulation is followed by the evaluation of the shortest path lengths. These are computed using Dijkstra’s shortest Path following [1,2,31]. Based on the results of the process simulation and the shortest path analysis, the evaluation criterion is calculated for all elements so that we obtain a point map with a qualitive measurement of the manufacturability for each element. In the next step, we use this point map to modify the structures built from the TO results. Finally, these two structures are merged into one geometry which represents a new design proposal with increased manufacturability.

These various steps of the workflow in Figure 1 are explained in more detail in the following subsections. The proposed workflows are exemplarily tested for HPDC and LPDC. Their simulation results and evaluation of manufacturability is then compared to unconstrained TO and manually redesigned parts.

### 2.1. Topology Optimization Setup

For TO, this article uses the software nTopology (Version 3.0.4, nTopology Inc., New York, NY, USA) and a density based TO method in combination with the popular solid isotropic material interpolation with penalization (SIMP) method [7] to solve a minimum compliance problem with a given volume constraint. In this TO method, the design variable $x_{i}$ for each element describes its relative density. Consequently, $x_{i}=1$ means the element is fully stressed and therefore seen as solid material. Accordingly, $x_{i}=0$ means that the element is not stressed and can be seen as a hole in this context. The TO problem can be written as

(3) $$\min C=\min u^{T}Ku,\;s.t.:\;v\;\leq \;\mu ,$$

where C denotes the compliance, K the stiffness matrix, and u describes the displacement vector. The volume constraint is written as relative volume v, which has to be equal or smaller than μ.

Since the availability of manufacturing constraints heavily varies between various TO programs and the objective here is to show the global improvement of manufacturability, manufacturing constraints are omitted. In general, the presented workflow can be conducted with an arbitrary TO program without any loss of performance.

For a testing example, we selected a cantilever beam as it is a well-known example in TO. The scope of our workflow is to turn an arbitrary initial design space into structural and process optimized design proposals for HPDC and LPDC. Therefore, we chose dimensions of 100 mm length and 50 mm width and height to see if our method leads to the small wall thickness, which is characteristic for HPDC, respectively, towards a structure that implies a directed solidification as desired for LPDC.

**Figure 2:**
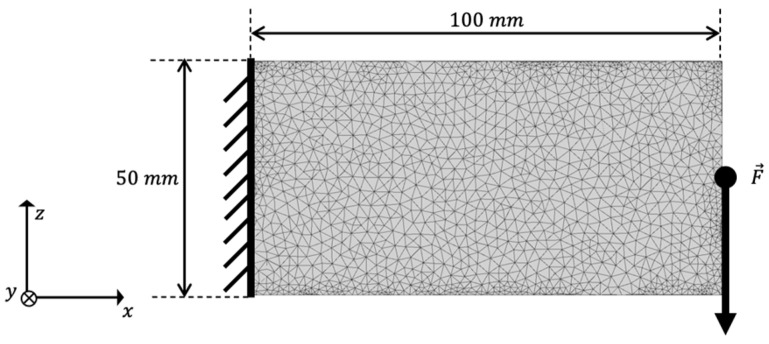
Setup of the cantilever beam used as benchmark example.

For the TO, the cantilever beam was loaded with a force of 100 N on the free end and the fixation of the cantilever is defined as a non-design space with a depth of 4 mm. The latter has been chosen to ensure that no element’s design variable of the fixation set is set to zero during the optimization iterations to maintain this area of the structure for later mounting of the part. The setup of the cantilever benchmark problem is given in Figure 2. The cantilever is meshed with linear tetrahedrons; the edge length is set to $\frac{Length}{4}$ mm.

For postprocessing of the TO step, a threshold limit for the design variable $x_{lim}$ is used. In this article, we set $x_{lim}$ to 0.6 for all TO runs. The TO results are then used to transform all elements whose design variable is greater or equal to the threshold into an implicit geometry. This implicit model is smoothed using a grid size of 1 mm and 3 iteration steps, re-meshed, and saved as structure optimized geometry. An implicit geometry model is analogously made from all elements whose design variable is less than $x_{lim}$.

**Figure 3:**
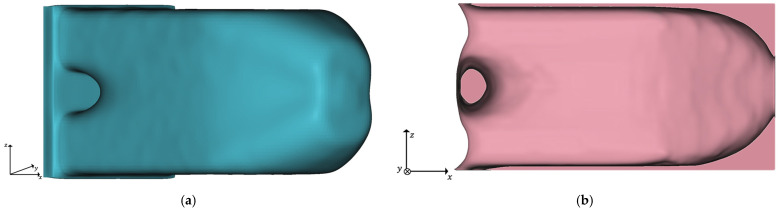
Results of the initial TO with λ = 0.2 and $x_{lim}$ = 0.6: (**a**) Implicit geometry of the optimized structure; (**b**) Cross-section of the implicit geometry of the TO-margin.

Consequently, we refer to this geometry as TO-margin, which consists of all elements of the design space that are not part of the structure optimized geometry. We use the TO-margin in the final postprocessing to add elements needed to increase the design proposal’s manufacturability. The optimized geometry and its TO-margin are shown exemplarily for the initial TO run in Figure 3.

### 2.2. Process Simulation and Evaluation

The PA we use in this work consists of a casting process simulation, followed by the shortest path analysis and the calculation of an evaluation criterion. For the simulation of the casting process, the commercial software Flow-3D (v5.0 Update 4, Flow Science Inc., Rottenburg, Germany) is used. This software calculates the fluid flow and solidification of the molten alloy using the FVM. The free surface between metal and air is defined by the VOF method [22].

Two different casting techniques (HPDC and LPDC) are evaluated as representation for large variations in the requirements of different casting processes. The simulations are built up separately for HPDC and LPDC and described shortly in the following. 

#### 2.2.1. High Pressure Die Casting

The filling simulation is particularly important for evaluating the TO results of the HPDC process. The molten metal is simulated as an incompressible fluid and the air in the mold is assumed to be an adiabatic gas. There is no flow calculation of the air. 

**Figure 4:**
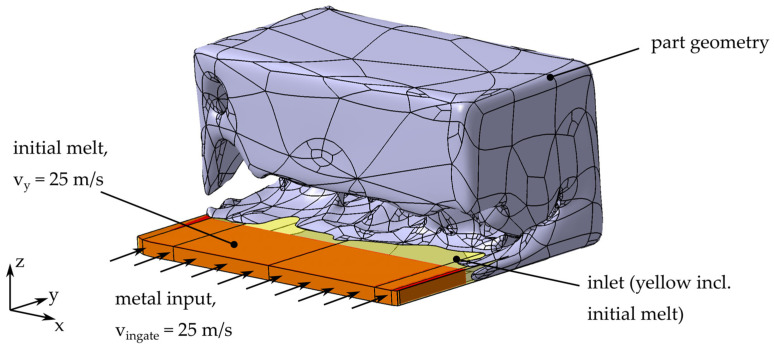
Simulation setup for HPDC, exemplarily shown for iteration *t* = 2. The surrounding die is not shown in the figure.

The simulation setup for the HPDC process is shown in Figure 4. For the initial iteration step, the part is defined as a rectangular solid with a length of 100 mm and a height and width of 50 mm. The part geometry of following iterations is defined by the results obtained by the TO. 

There are several options for placing the ingate points for this geometry. On the basis of the volume fraction weight sums of the geometry evaluated by the shortest-path algorithm [2], we decided to place the ingate along the longest edge of the initial design space. The ingate has a height of 4 mm and the ingate velocity is defined as 25 $\frac{m}{s}$. At the transition between ingate and the part geometry, even small edges lead to splashing. This splashing causes a more difficult evaluation of the process. For reducing this misinterpretation due to splashing at the sharp edges, the transition between ingate and part volume is smoothed in every iteration step. The ingate outside of the initial part volume is set to be filled with an aluminum alloy melt at *t* = 0 with a homogeneous velocity of 25 $\frac{m}{s}$ along the y-axis. Vents as well as overflows are not considered, because they result from the final part and a placement without knowledge of the final part geometry is not reasonable. The casting mold is defined by a sufficiently big surrounding box. For the mesh a cell size of 1 mm is used.

The material used in the HPDC process is aluminum A380 (AlSi9Cu3). The material parameters are listed in the Supplementary Materials (Table S1). A casting temperature of 675 °C prevents solidification of the melt during the mold filling simulation; thermal effects can therefore be neglected.

**Figure 5:**
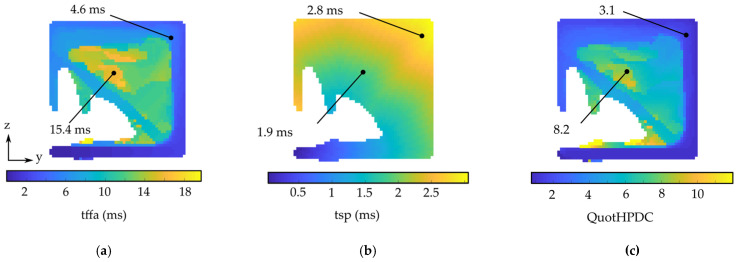
Evaluation of the filling simulation of the HPDC process. The simulation results are shown for a cross section for a part (HPDC simulation, iteration *t* = 2) during the iterative optimization process. A low quotient indicates cells with a good filling capacity. (**a**) Time of first fluid arrival, *tffa*; (**b**) Time for shortest path length, *tsp*; (**c**) Quotient of *tffa* and *tsp*, QuotHPDC; For better clarity, the maximum legend is set to 12, although there are a few cells with a value of QuotHPDC up to 35.

Since the flow lengths in a HPDC part have to be as short as possible [16], we decided to use a modified quotient introduced by [1] as a quality criterion of the process. We use the quotient (*QuotHPDC*) of the time of first fluid arrival (*tffa*) and the theoretical time of arrival along the shortest path (*tsp*) for every volume cell. Figure 5 shows the comparison of the *tffa*, *tsp* and *QuotHPDC* criteria for a cross section of an optimized beam. First, *tffa* is calculated in Flow3D. Then, a graph is generated from the finite volume mesh. In this graph, each FVM cell (voxel) is represented as nodes. Their position is defined by the mid points of each voxel. Subsequently, links for every voxel to its next nodes in its immediate neighborhood are created. In our work, we use 26-connectedness, with 6 face neighbors, 12 edge neighbors and 8 vertex neighbors. The weight of every link is defined by its Euclidean distance between the mid points of two neighboring voxels. In this graph, the shortest path is computed with the closest points of the ingate to the part as starting points of the algorithm. The time for shortest path (*tsp*) is defined as the shortest path length divided by a constant ingate velocity. Finally, the QuotHPDC criterion is calculated as the following:

(4) $$QuotHPDC=\frac{tffa}{tsp}$$

In this context, a high *QuotHPDC* means that the melt reaches the cell over a large flow path length, although the direct path between ingate and cell is shorter. Therefore, a low *QuotHPDC* is desirable. A *QuotHPDC* of 1 means that the melt reaches the cell via the shortest path and thus represents the optimum for the casting process.

#### 2.2.2. Low Pressure Die Casting

In our work, we focus on the challenge to design a part ensuring a directed solidification path to the raiser tube to avoid shrinkage defects and macro porosity in LPDC parts. Therefore, a filling simulation and a subsequent solidification simulation are required for the evaluation of the LPDC process. The temperature distribution at the end of the filling simulation is considered as a boundary condition in the solidification simulation. In the real process, solidification shrinkage and shrinkage in solid state both lead to voids in areas that are insufficient fed. These areas are represented in the simulation by empty cells, with no results for the solidification time. For the TO, a quality criterion of the process is needed that allows a qualification of all areas in the part. Therefore, in this work, the shrinkage is not modeled in the simulation to avoid empty cells.

**Figure 6:**
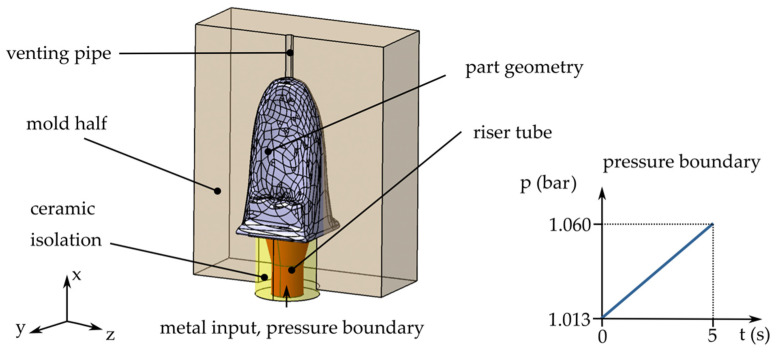
Simulation setup for LPDC, exemplarily shown for iteration *t* = 4. The pressure boundary is defined by a linear pressure increase on the bottom of the riser tube.

The optimization for the LPDC process starts with the same initial volume as in the HPDC. Further geometries are derived by the TO. The longest edge is placed vertically along the x-axis. The simulation setup is shown exemplarily in Figure 6.

The cavity is filled via a filling tube at the bottom of the component. The ceramic feeder tube measures 20 mm in diameter at the bottom and widens to 30 mm at the top. The geometry is placed in the center of a cuboid mold made of H-13 steel with external dimensions of 130 mm × 100 mm × 180 mm. Venting of the cavity is ensured by a venting pipe at the top of the component. The filling simulation terminates as soon as the melt reaches this pipe. The gravity force of 9.81 $\frac{m}{s^{2}}$ is defined along the negative x-direction. 

The metal input is defined as a pressure boundary condition. The pressure curve is defined as a linear function starting at an initial pressure p_0_ of 1.013 bar at *t* = 0 s and rising to a maximum pressure of 1.060 bar at *t* = 5 s. The maximum pressure was estimated using the formula for hydrostatic pressure for a fully filled cavity:

(5) $$p=p_{0}+\rho gh,$$

where, respectively, p denotes the pressure, $p_{0}$ denotes the initial pressure, g denotes the acceleration due to gravity, and h describes the height in the x-direction.

For the LPDC process, the aluminum alloy A356 (AlSi7Mg0.3) is used. The material properties are listed in the Supplementary Materials (Table S2). The casting temperature is defined as 708 °C. The defined heat transfer coefficients for the LPDC simulation are listed in the Supplementary Materials (Table S3).

For the LPDC process, a directed solidification path towards the filling tube is desired. To reach this goal, we propose the use of the logarithmic quotient (*QuotLPDC*) of the solidification time ($t_{sol}$) and the distance between the evaluated cell mid-point and the ingate level along the x-axis (h), which is calculated by

(6) $$QuotLPDC=\log{\left(\frac{t_{sol}}{h}\right)}_{.}$$

As solidification time for a specific voxel, we use the maximum solidification time of all voxels in one yz-level. In this way, all cells that lead to thick wall thicknesses in the yz-level are rated with the resulting high solidification time in this level. A high value of *QuotLPDC*, with a short distance to the filling tube and a high solidification time, marks cells which are favorable from the casting point of view. These promising cells are not removed by the TO until sufficiently low solidification times are achieved in farther cells. This results in a tapering of the component with increasing *h* and ensures feeding during the solidification process. We note that this quality criterion is only valid if the flow lengths and the distance to the ingate level along the x-axis correlate.

### 2.3. Post-Processing

**Figure 7:**
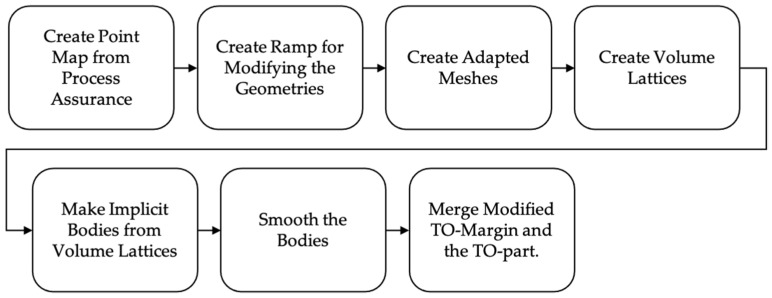
Postprocessing Workflow.

The objective for the final postprocessing is to remove a volume fraction in the amount of λ from the design space and then add and or remove some additional material from the TO-margin to improve the manufacturability. This is done with nTopology’s implicit modeling technology [34] and the corresponding workflow is illustrated in Figure 7. The implicit modeling technology provides the big advantage that structures are described by equations and not by edges as in the boundary representations. This means that various structures can be merged together easily by using Boolean operators. 

**Figure 8:**
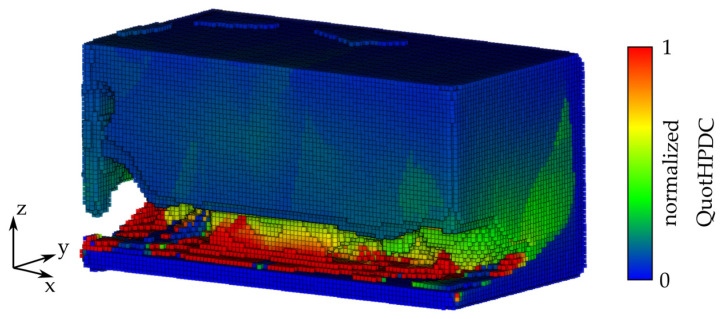
Exemplary point map for the *QuotHPDC* in iterative high-pressure die casting simulation, iteration *t* = 2.

At the beginning, there is a point map, as illustratively shown in Figure 5, with the used evaluation criterion from the process assurance. Here, the criterion *QuotHPDC* (Figure 8) is applied to modify the high pressure die casting part, respectively, and *QuotLPDC* modifies the low pressure die casting part. To avoid a falsification in the further post-processing, outliers of the minimum one percent and the maximum one percent of the criterion value are removed. The criteria value of these cells is set to the minimum or maximum of the remaining values of the quality criteria. Afterwards, the quality criteria are rescaled in a range between 0 and 1 following:

(7) $$x_{i}{}^{\prime }=\frac{x_{i}-\min\left(x\right)}{\max\left(x\right)-\min\left(x\right)}$$

**Figure 9:**
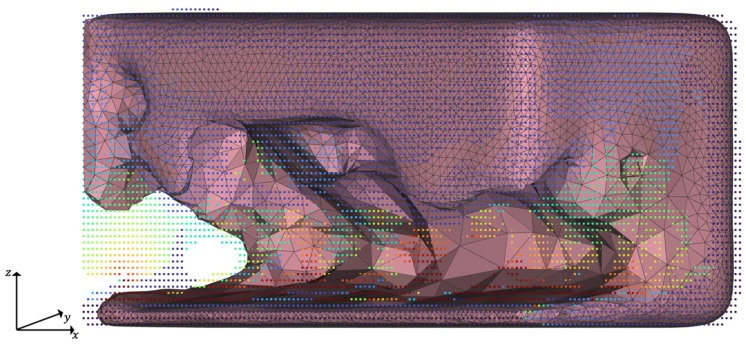
Creation of adapted meshes of the TO-margin by example of iterative HPDC optimization, iteration *t* = 5. In the adapted tetrahedral mesh, the edge lengths are controlled by the corresponding *QuotHPDC* ramp function.

The rescaled map is then used to define two linear ramp functions for modifying the optimized structure and the TO-margin. Therefore, for each ramp, a lower and an upper limit for weighting the evaluation criterion are selected. These ramps are then used to adapt the edge lengths of the meshes from which a volume lattice is created; see Figure 9.

**Figure 10:**
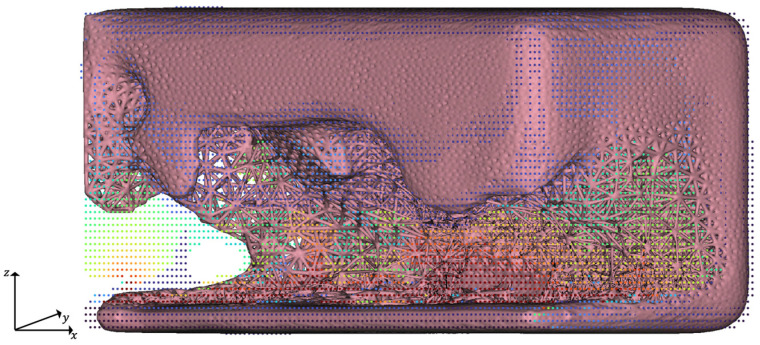
Implicit volume mesh of the TO-margin before smoothing by example of iterative high-pressure casting optimization, iteration *t* = 5. The strut thickness of the volume lattice is controlled by *QuotHPDC*.

In the next step, the volume mesh is transferred into an implicit body, and the thickness of the mesh is thereby also controlled by the evaluation criterion ramp. To delete undesired elements from the part, which are characterized by thin connections, a smoothing step is applied. In this case, the grid size was 5 mm, and 5 smoothing iterations were conducted. The implicit volume lattice body before smoothing is shown representatively in Figure 10.

**Figure 11:**
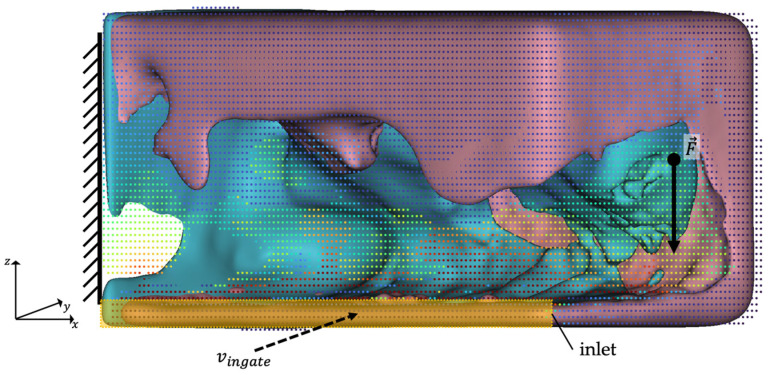
Final result of the postprocessing workflow by example of iterative high-pressure casting optimization, iteration *t* = 5. The modified TO-margin (red) and the modified TO-part (blue) are merged into one body which represents the new design proposal. For a better understanding of the result, the boundary conditions of TO and filling simulation are in auxiliary shown.

The last step merges the modified bodies for the optimized geometry and the TO-margin; finally, the new design proposal is meshed and prepared as input for the next iteration; see Figure 11.

### 2.4. Evaluation and Metric of the Results

The results are analyzed regarding manufacturability and structural performance. Thereby, the structural performance is measured by conducting a finite element analysis and evaluating the relative-volume-related stiffness $S_{VR}$, which is given by

(8) $$S_{VR}=\frac{F}{u_{max}}\;\frac{1}{v},$$

where F and $u_{max}$ are, respectively, the force and the resulting maximum displacement, and v denotes the relative volume of the tested design proposal. The settings for the finite element analysis were identical to the settings of the TO shown in Figure 2.

For determining the improvement of manufacturability qualitatively, the median of the respective evaluation criterion is evaluated for both cases, the HPDC and LPDC simulations.

To analyze the results in detail, we also look on the evaluation criterion of every finite volume cell in the design proposal. Consequently, a part is supposed to be manufacturable if the evaluation criterion is in the desired interval for many cells. We refer to these cells further as manufacturable elements θ, which are the relative sum of the fraction of cells in a given interval

(9) $$\theta =\frac{\sum x_{lower}\;\leq x_{i}\leq \;x_{upper}}{\sum x_{i}},$$

where $x_{lower}$ and $x_{upper}$ are, respectively, the lower and upper bond of the interval. The lower and upper bonds we used in this article are listed in Table 1. While for HPDC the absolute values of *QuotHPDC* were taken, we took the normalized values of *QuotLPDC* due to its formulation of and physical meaning.

## 3. Results

The initial design space (DS) from the TO setting in Figure 2 is used as the reference model, and additionally, eight other configurations were evaluated. This led to nine configurations in total including the One-Step (OS) and iterative (IT) optimization approaches, and as reference solutions, solely TO, respectively, manually process optimized geometries. An overview of all configurations with their settings is given in Table 2.

The step-length controls the target volume constraint of the TO. During the post-processing, elements are added or eliminated based on the PA results, affecting the step-length. In particular cases, the effective step-length after post-processing ranged from 5% to 10% for HPDC and 7% to 21% for LPDC.

### 3.1. One Step Optimization

**Figure 12:**
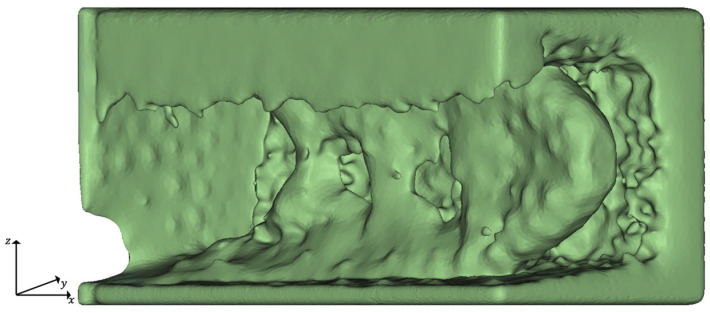
Result of the HPDC-OS Optimization, with a volume of 50% compared to the initial design space.

The optimization results of the HPDC-OS configuration are shown in Figure 12. The geometry can be described by a U-profile caused by the fluid flow of the filling simulation. The struts along the midplane are resulting from the TO. Additionally, the result indicates that the position of the ingate system has a significant influence on the final geometry. It can be seen that the automated smoothing procedure of the global postprocessing did not work perfectly on this example and should be adapted for further optimizations.

**Figure 13:**
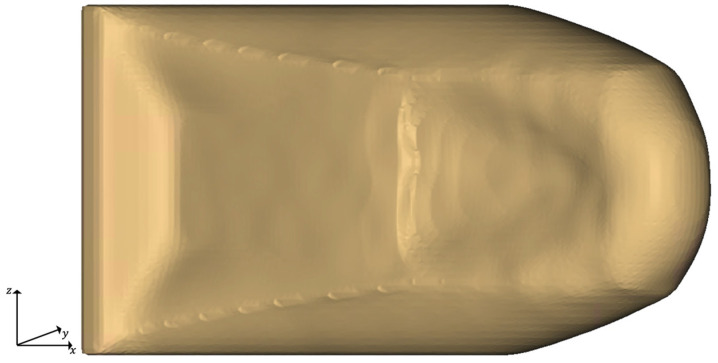
Result of the LPDC-OS Optimization, with a volume of 49% compared to the initial design space.

The LPDC-OS geometry represents a beam tapering towards the load point. Adversely, the smoothing worked well on the LPDC-OS configuration as shown in Figure 13.

Both results differed significantly from each other, indicating that the evaluation criteria developed were leading to design proposals, which are dedicated, respectively, to HPDC and to LPDC. One advantage of the One-Step approach is that the process simulation must run only once, and thus, the process is very time efficient.

### 3.2. Iterative Optimization

**Figure 14:**
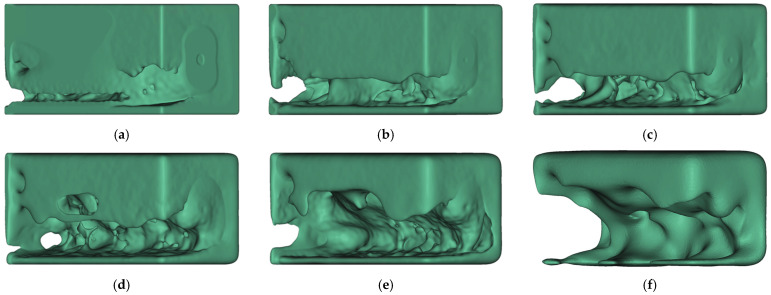
Result of each iteration of the Iterative Optimization of HPDC parts for λ = 0.2; the volume of the design proposals is given in brackets: (**a**) HPDC-IT_1 (90%); (**b**) HPDC-IT_2 (85%); (**c**) HPDC-IT_3 (76%); (**d**) HPDC-IT_4 (68%); (**e**) HPDC-IT_5 (60%); (**f**) HPDC-IT_6 (50%).

Analogously, the Iterative Optimization workflow was also tested on the cantilever beam for HPDC and LPDC processes. The results for HPDC-IT are presented in Figure 14; each iteration is displayed separately.

The used termination criterion was the applied volume constraint of 50%, which was reached after six iterations. It can be seen that the design space is continuously hollowed with each iteration and tends to develop the shape of a U-profile. For each iteration, about 10% of material was added to the TO-structure as a trade-off of the manufacturability.

**Figure 15:**
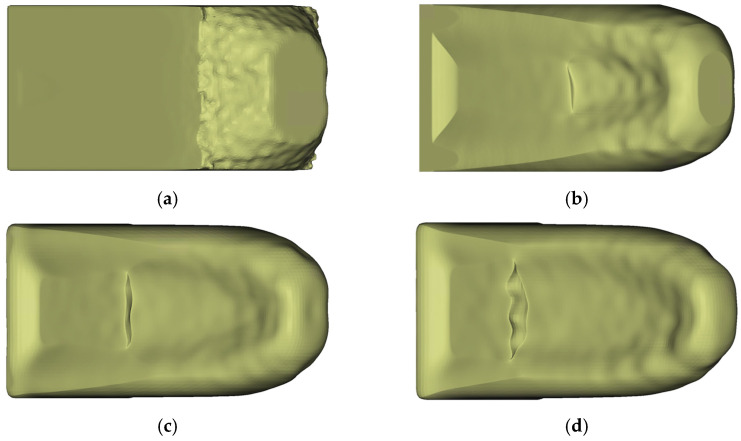
Result of each iteration of the Iterative Optimization of LPDC parts for λ = 0.2; the volume of the design proposals is given in brackets: (**a**) LPDC-IT_1 (93%); (**b**) LPDC-IT_2 (72%); (**c**) LPDC-IT_3 (60%); (**d**) LPDC-IT_4 (47%).

The LPDC-IT optimization reached the termination criterion after four iterations, as shown in Figure 15. As for the HPDC-IT results, each iteration is displayed separately to visualize the iterative progress of the optimization procedure.

### 3.3. Comparison of the Approaches

To show the differences between the obtained design proposals, the results of the approaches are compared with each other and benchmark models in terms of manufacturability and stiffness.

#### 3.3.1. Manufacturability Evaluation

**Figure 16:**
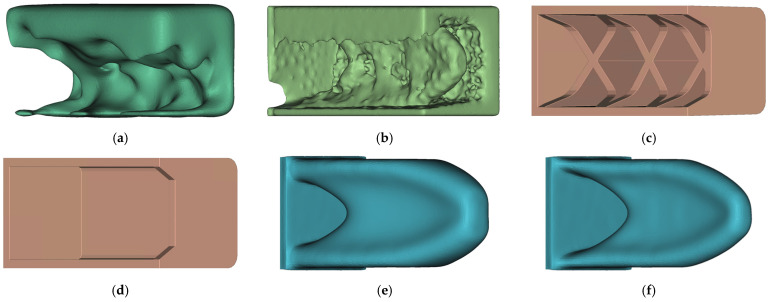
Comparison of all HPDC and TO design proposals: (**a**) HPDC-IT_6; (**b**) HPDC-OS; (**c**) HPDC-50; (**d**) HPDC-30; (**e**) TO-50; (**f**) TO-30.

In Figure 16, the final design proposals of HPDC-IT and HPDC-OS are shown next to the four reference solutions. The manually created reference models HPDC-50 and HPDC-30 were modelled as a U-profile with a wall thickness of 4 mm. On the side of the fixation, as well, on the force-induced side, cross walls were placed. For the HPDC-50 geometry, additionally supporting struts were placed. 

**Figure 17:**
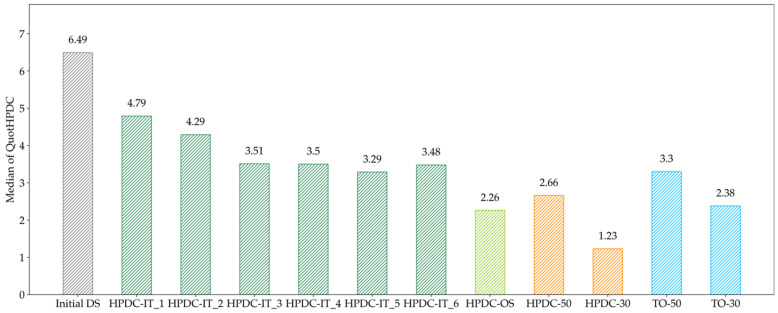
Evaluation of the median of QuotHPDC for all optimized geometries. The evaluation indicates that the iterative approach gets trapped in a local minimum.

The HPDC-OS design proposal looks like a combination of HPDC-30 and TO-50, whereas the HPDC-IT also forms a U-profile but no supports along the mid axis. The medians of QuotHPDC for all design proposals in Figure 14 as well as Figure 16 are presented in Figure 17.

**Figure 18:**
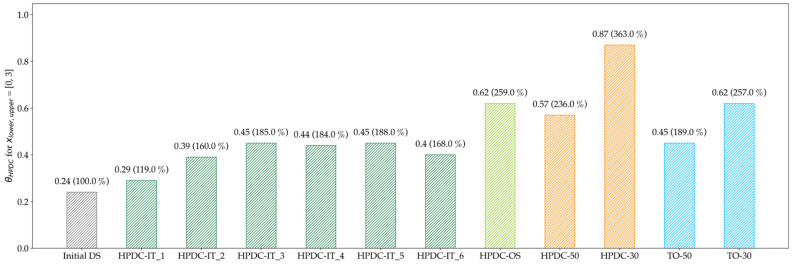
Evaluation of the manufacturability of HPDC design proposals; the deviation compared to the Initial DS is given in brackets. The One-Step optimization approach increases the manufacturability of the design proposal by 159%.

The median decreased continuously for the first five iteration and seemed to find a minimum at HPDC-IT_5. Nevertheless, the HPDC-OS median is significantly lower, which indicates that the HPDC-IT approach is trapped in local minima for the chosen step-length of 0.2. The results of the evaluation of the number of manufacturable elements in Figure 18 further support this assumption.

Compared to the Reference, the manufacturability increased by, respectively, 68% for HPDC-IT and 159% for HPDC-OS. Additionally, here, the improvement is decreased for the last iteration in HPDC-IT.

It is to state globally that both approaches led to an improvement of the manufacturability but different design proposals. 

**Figure 19:**
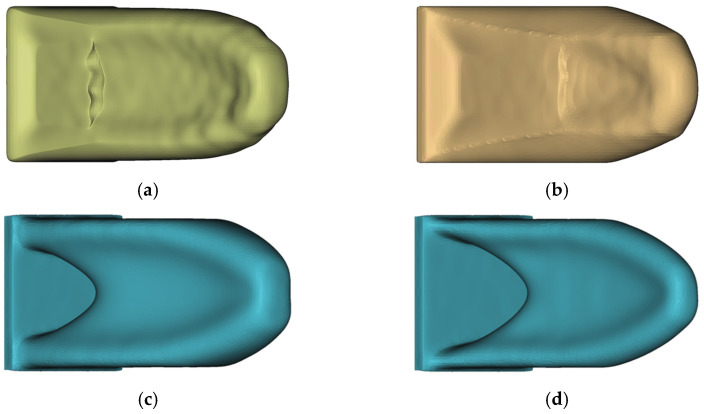
Comparison of all LPDC and TO design proposals: (**a**) LPDC-IT_4; (**b**) LPDC-OS; (**c**) TO-50; (**d**) TO-30.

By looking at the LPDC-IT design proposals in Figure 19, one can first detect that the results for LPDC-IT and LPDC-OS had a high similarity.

**Figure 20:**
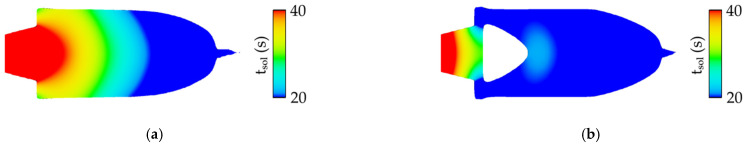
Solidification time for a cross section along the mid-plane. In the geometry LPDC-IT_5, a directed solidification was achieved, whereas for TO-30, a hot spot above the void region could not be fed adequately. (**a**) LPDC-IT_5; (**b**) TO-30.

**Figure 21:**
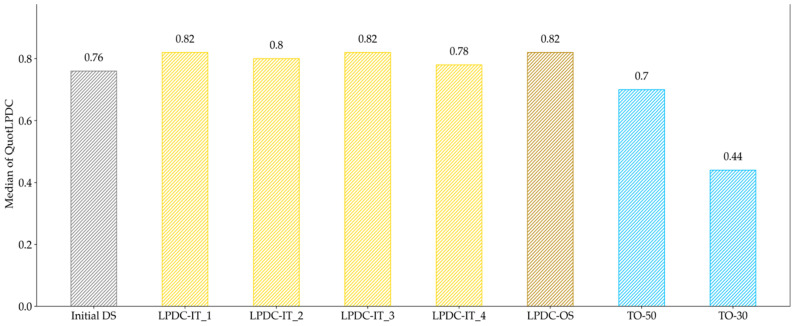
Evaluation of the median of *QuotLPDC* for all investigated geometries in the LPDC process. The median hardly changes between the design of the iteration and the One-Step result.

The main difference to the TO design proposals is that the void in the lower region of the design space is filled based on the results of the casting process simulation. This is supposed to be beneficial for obtaining a directed solidification path, which can be confirmed by evaluation of the solidification time in Flow3D in Figure 20 as well as in the evaluation of *QuotLPDC* in Figure 21.

**Figure 22:**
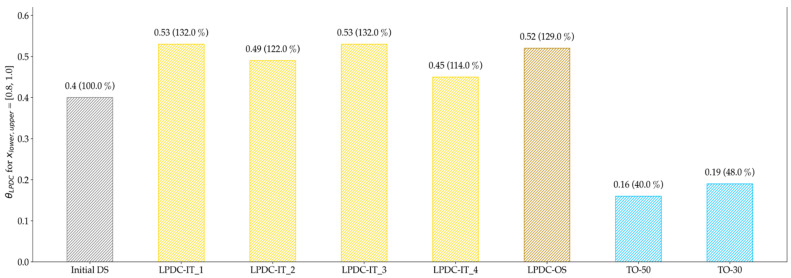
Evaluation of the manufacturability of LPDC design proposals; the deviation compared to the Initial DS is given in brackets. The evaluation shows that the TO configurations have a significantly lower number of manufacturable elements for LPDC.

The median for the various design proposals was on a similar level, except for the TO results, which are significantly lower. By evaluating the manufacturable elements in Figure 22, it is clearly observable that the TO design proposals are less suited for manufacturing. Besides this, no clear tendency regarding convergence towards a specific solution can be seen from this point of evaluation.

#### 3.3.2. Structural Analysis

**Figure 23:**
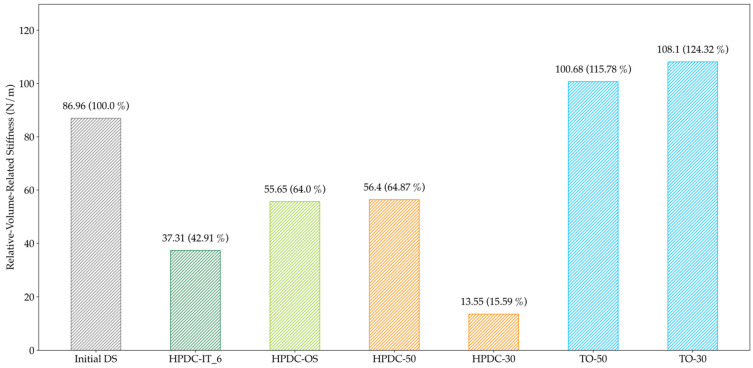
Volume-related stiffness for all HPDC configurations; the deviation to the Initial DS is noted in brackets. The One-Step and iterative HPDC configuration show significantly lower stiffness than the TO configurations.

**Figure 24:**
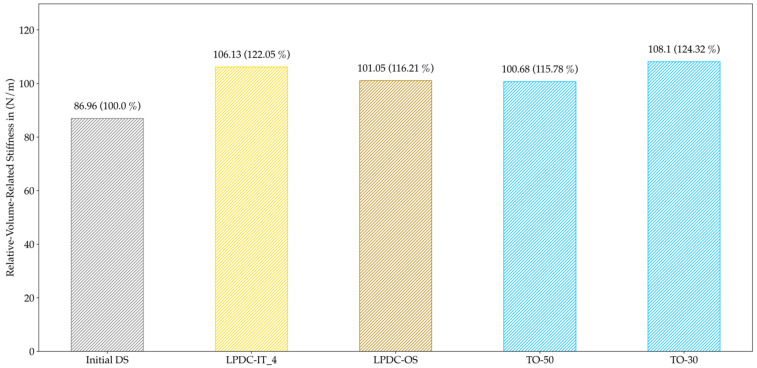
Volume-related stiffness for all LPDC configurations; the deviation to the Initial DS is noted in brackets. Both approaches show similar relative-volume-related stiffness to the TO configurations.

The volume-related stiffness is presented in Figure 23 for all HPDC configurations and in Figure 24 for all LPDC configuration.

For HPDC, the TO design proposals clearly outperformed the other results, while HPDC-OS was equivalent to the manually designed HPDC-50. Nevertheless, for LPDC, both approaches led to results slightly better than TO-50 and close to TO-30. As a result, a new geometry has been created which offers better castability while maintaining the high specific stiffness of the component.

## 4. Discussion

This article’s objective was to combine TO and PA results to obtain structure- and process-optimized design proposals. The combination of the two results was done by using the implicit modeling engine of nTopology [34]. The results of TO are taken in the form of the most stressed elements, which are identified by a value of the design variable close to 1. Since the availability of casting manufacturing constraints is strongly dependent on the used software, we used a mainly unconstrained TO setup so that our framework is potentially applicable to every commercial TO software. This approach eliminates manual work in redesigning parts and leads to design proposals that can be used directly for PA, saving a significant amount of time in the highly iterative process of developing optimized cast parts.

For evaluating the PA by means of a casting process simulation in HPDC processes, the criterion proposed in [1] was further developed to describe the quotient of the time of first fluid arrival and the theoretical time of arrival along the shortest path for every volume cell. By applying this evaluation criterion, an increase of manufacturable elements compared to the initial design space of 68% for HPDC-IT and 159% for HPDC-OS was obtained. These improvements show better performance than the results in [1], where the distribution of the distance travelled by fluid divided by the shortest path was minimized by manual redesigning of the design proposal and achieved an improvement of circa 15%. Additionally, the structure of the design proposals clearly differed. It is assumed that the smaller step-length used in HPDC-IT led to a local minimum at HPDC-IT_5. Consequently, the step-length is said to be a major parameter for adjusting the performance as well as the time of the overall optimization process. In addition to the step-length, the various smoothing parameters and ramp settings for reconstructing a design proposal from the simulation results also have an impact on the design, which should be investigated in future work. Since the TO objective function was a minimum compliance problem, the volume-related stiffness was used to compare the configurations. As shown in Figure 22, HPDC-OS was equivalent to the manually designed part HPDC-50 but clearly outperformed by solely topology optimized configurations. This indicates that the increased manufacturability causes a trade-off in the structural performance. On the other hand, the process simulation showed a high dependency on the initial design volume. Further process simulations with differing design volumes could lead to new local maxima in the optimization process, which would improve the trade-off.

Hence, the restrictions in LPDC processes strongly differ from HPDC processes so that a second evaluation criterion for PA was needed. Due to the fact that a directed solidification path is desired in LPDC, the evaluation criterion for LPDC (*QuotLPDC*) was based on the solidification time and its level in the direction in which solidification should occur. For the chosen cantilever problem, this direction was identical with the z-axis, so that the description of a solidification axis was easily possible. Nevertheless, for more complex models, it is important to find good solutions for how to describe the desired solidification axis. The results instead showed that the number of manufacturable elements did not change much during the optimization, leading to an increase of 14% for LPDC-IT and 29% for LPDC-OS. This is due to the fact that the initial design space is much better suited for LPDC than for HPDC in general. By looking at the medians of LPDC configurations in Figure 19, no clear tendency of convergence for LPDC-IT can be detected, although all geometries allowed a directed solidification towards the raiser tube. In this case, LPDC-OS shows also a slightly better performance. Thereby, we indicate that larger step-lengths may lead to better results in HPDC as well as in LPDC optimizations. The comparison in Figure 20 shows that the proposed method avoids areas which occur in solely TO-configurations and that cannot be fed properly. This enhancement is equivalent to the results presented in [3,4], where the incorporation of process knowledge also led to improved solidification behavior compared to a TO without process knowledge. Adversely to the HPDC results for structural evaluation in Figure 23, both LPDC-IT and LPDC-OS can be seen as comparable to the solely topology optimized configurations, and thus represent structure- and process-optimal design proposals.

To summarize these results globally, it can be stated that the combination of TO and PA by implicit modeling based on a geometry-based evaluation criterion led to an improvement in the manufacturability of the design proposals. Combining TO and PA results and the construction of the new design proposal is automated and done in less than 10 min, which is a significant time saving compared to manual reconstruction. Compared to previous work in the field, the proposed method leads to better, respectively equivalent results, while using computationally cheaper information. This indicates that the geometry-linked evaluation criteria have sufficient information to describe the manufacturability of design proposals qualitatively and can therefore be used to replace the process simulation during the TO iterations. Since the process simulations were time extensive, about 30 min for HPDC and 180 min for LPDC, the replacement of process simulations by computationally cheaper metamodels is the consequent next step in our research to significantly enhance the performance of our framework. 

By evaluating all HPDC design proposals, no configuration was found that shows high manufacturability and high mechanical performance. This observation assumes that the parameters or the design space were not optimally chosen for this problem. On the other hand, the LPDC design proposals showed that geometries that are both structure- and process-optimal could be identified by the proposed method. These results indicate that the combination of PA and TO leads to a strong trade-off either in manufacturability or structural performance, if the optimization setup is not well-suited for the considered casting process. For instance, a small well thickness is characteristic for HPDC parts, and this could be achieved by choosing a lower target volume constraint for TO.

We also identified the step-length and the design space in combination with the settings for reconstructing the geometry as critical parameters affecting the solution. An extensive study of the influence of these parameters must be conducted in future work.

One major bottleneck for performing parameter studies with casting process simulations as a part of the PA is the time consumption of the casting process simulation and the data transfer between the TO-software and the casting process simulation software. Thus, the development of a physical metamodel is desired to obtain high-fidelity values of the evaluation criteria without performing a casting process simulation. The development of these metamodels for HPDC and LPDC processes is crucial for performing efficient parameter studies and, therefore, the most important subject of future work.

## 5. Summary and Outlook

This article showed a method for combining TO and PA with implicit modeling and geometry-based evaluation criteria. Thereby, the automation of the reconstruction of new design proposals can accelerate the overall development process of cast parts. Additionally, the flexible description of the workflow allows its adaption to arbitrary TO problems. The proposed method was tested on a cantilever problem for HPDC and LPDC processes compared to manually designed and solely topology optimized design proposals. We showed that the approach leads to design proposals with improved manufacturability and identified critical parameters influencing the solutions. Since we try to solve a multi-objective optimization problem, increasing manufacturability is likely to provoke a trade-off in mechanical performance. Consequently, an extensive study of the critical parameters’ impact on the design proposals is necessary for future work, developing good default settings for problem classes and controlling the trade-off between the structural- and process-optimal solution. In this context, the development of physical metamodels to obtain the evaluation criteria can reduce the time effort for PA immensely.

## Figures and Tables

**Table 1:** Upper and lower bounds for evaluating the manufacturable cells.

	$x_{lower}$	$x_{upper}$
HPDC	0.0	3.0
LPDC	0.8	1.0

**Table 2:** Overview of the selected configurations. The optimization approach differs between the workflows for, respectively, One-Step, iterative optimization, TO, and manual part design. The target volume is given as relative volume. For the iterative optimization, the step-length in the TO is additionally given in brackets.

Configuration Name	Optimization Approach	Target Volume (Step-Length)
Initial DS	None	1
HPDC-OS	One-Step	0.5
HPDC-IT	Iterative	0.5 (0.2)
HPDC-50	Manual	0.5
HPDC-30	Manual	0.3
LPDC-OS	One-Step	0.5
LPDC-IT	Iterative	0.5 (0.2)
TO-30	TO	0.3
TO-50	TO	0.5

## Data Availability

The data presented in this study are available on reasonable request from the corresponding author.

## Supplementary Materials

The following are available online at https://www.mdpi.com/article/10.3390/ma14133715/s1.

## References

[1] Hautsch S., Heilmeier F., Rieg F., Volk W., Binz H., Bertsche B., Spath D., Roth D. (2017). Effiziente Entwicklung von prozessoptimalen druckgussbauteilen durch kombination von topologieoptimierung und prozesssimulation. Stuttgarter Symposium für Produktentwicklung SSP17.

[2] Heilmeier F., Goller D., Opritescu D., Thoma C., Rieg F., Volk W. (2016). Support for Ingate Design by Analysing the Geometry of High Pressure Die Cast Geometries Using Dijkstra’s Shortest Path Algorithm. Adv. Mater. Res..

[3] Franke T., Fiebig S., Paul K., Vietor T., Sellschopp J., Schumacher A., Vietor T., Fiebig S., Bletzinger K.-U., Maute K. (2018). Topology optimization with integrated casting simulation and parallel manufacturing process improvement. Advances in Structural and Multidisciplinary Optimization.

[4] Franke T., Fiebig S., Bartz R., Vietor T., Hage J., vom Hofe A., Rodrigues H.C., Herskovits J., Mota Soares C.M., Araújo A.L., Guedes J.M., Folgado J.O., Moleiro F., Madeira J.F.A. (2019). Adaptive Topology and Shape Optimization with Integrated Casting Simulation. EngOpt 2018, Proceedings of the 6th International Conference on Engineering Optimization, Braunschweig, Germany, 5–9 June 2017.

[5] Glamsch J., Deese K., Rieg F. (2019). Methods for Increased Efficiency of FEM-Based Topology Optimization. Int. J. Simul. Model..

[6] Harzheim L. (2019). Strukturoptimierung Grundlagen und Anwendung.

[7] Rosnitschek T., Hentschel R., Siegel T., Kleinschrodt C., Zimmermann M., Alber-Laukant B., Rieg F. (2021). Optimized One-Click Development for Topology-Optimized Structures. Appl. Sci..

[8] Harzheim L., Graf G. (2005). A Review of Optimization of Cast Parts Using Topology Optimization: I—Topology Optimization without Manufacturing Constraints. Struct. Multidisc. Optim..

[9] Harzheim L., Graf G. (2006). A Review of Optimization of Cast Parts Using Topology Optimization: II-Topology Optimization with Manufacturing Constraints. Struct. Multidisc. Optim..

[10] Vatanabe S.L., Lippi T.N., de Lima C.R., Paulino G.H., Silva E.C.N. (2016). Topology Optimization with Manufacturing Constraints: A Unified Projection-Based Approach. Adv. Eng. Softw..

[11] Gersborg A.R., Andreasen C.S. (2011). An Explicit Parameterization for Casting Constraints in Gradient Driven Topology Optimization. Struct. Multidisc. Optim..

[12] Sato Y., Yamada T., Izui K., Nishiwaki S. (2017). Manufacturability Evaluation for Molded Parts Using Fictitious Physical Models, and Its Application in Topology Optimization. Int. J. Adv. Manuf. Technol..

[13] Li Q., Chen W., Liu S., Fan H. (2018). Topology Optimization Design of Cast Parts Based on Virtual Temperature Method. Comput. Aided Des..

[14] Wang Y., Kang Z. (2017). Structural Shape and Topology Optimization of Cast Parts Using Level Set Method: Structural Shape and Topology Optimization of Cast Parts Using Level Set Method. Int. J. Numer. Meth. Eng..

[15] Xu B., Han Y.S., Zhao L., Xie Y.M. (2019). Topology Optimization of Continuum Structures for Natural Frequencies Considering Casting Constraints. Eng. Optim..

[16] Nogowizin B. (2011). Theorie und Praxis des Druckgusses.

[17] Cuesta R., Delgado A., Maroto A., Mozo D. (2006). Numerically modeling oxide entrainment in the filling of castings: The effect of the webber number. J. Oper. Manag..

[18] Kwon H.-J., Kwon H.-K. (2019). Computer Aided Engineering (CAE) Simulation for the Design Optimization of Gate System on High Pressure Die Casting (HPDC) Process. Robot. Comput. Integr. Manuf..

[19] Dou K., Lordan E., Zhang Y.J., Jacot A., Fan Z.Y. (2020). A Complete Computer Aided Engineering (CAE) Modelling and Optimization of High Pressure Die Casting (HPDC) Process. J. Manuf. Process..

[20] Mehtedi M.E., Mancia T., Buonadonna P., Guzzini L., Santini E., Forcellese A. (2020). Design Optimization of Gate System on High Pressure Die Casting of AlSi13Fe Alloy by Means of Finite Element Simulations. Procedia CIRP.

[21] Shahane S., Aluru N., Ferreira P., Kapoor S.G., Vanka S.P. (2019). Finite Volume Simulation Framework for Die Casting with Uncertainty Quantification. Appl. Math. Model..

[22] Hirt C.W., Nichols B.D. (1981). Volume of fluid (VOF) method for the dynamics of free boundaries. J. Comput. Phys..

[23] Cleary P., Ha J., Alguine V., Nguyen T. (2002). Flow modelling in casting processes. Appl. Math. Model..

[24] Dabade U.A., Bhedasgaonkar R.C. (2013). Casting Defect Analysis using Design of Experiments (DoE) and Computer Aided Casting Simulation Technique. Procedia CIRP.

[25] Hahn I., Sturm J. (2015). Von der Simulation zur gießtechnischen Optimierung. Giesserei.

[26] Hahn I., Sturm J. Autonomous optimization of casting processes and designs. Proceedings of the World Foundry Congress.

[27] Jadhav A.R., Hujare D.P., Hujare P.P. (2021). Design and Optimization of Gating System, Modification of Cooling System Position and Flow Simulation for Cold Chamber High Pressure Die Casting Machine. Mater. Today Proc..

[28] Pinto H.A., Silva F.J.G., Martinho R.P., Campilho R.D.S.G., Pinto A.G. (2019). Improvement and Validation of Zamak Die Casting Moulds. Procedia Manuf..

[29] Shahane S., Aluru N., Ferreira P., Kapoor S.G., Vanka S.P. (2020). Optimization of Solidification in Die Casting Using Numerical Simulations and Machine Learning. J. Manuf. Process..

[30] Liu J., Ma Y. (2016). A Survey of Manufacturing Oriented Topology Optimization Methods. Adv. Eng. Softw..

[31] Dijkstra E.W. (1959). A note on two problems in connexion with graphs. Numer. Math..

[32] OPTIMAT: Entwicklung und Validierung von Softwaretools zur Simulation des Betriebsverhaltens von Werkstoffen in thermisch und Mechanisch Hoch Belasteten Komponenten, Arbeitsanteil NEMAK Wernigerode GmbH: Abschlussbericht, Berichtszeitraum: 1 May 2007–31 October 2010. Werkstoffinnovation für Industrie und Gesellschaft—WING: 2010. https://www.tib.eu/de/suchen?tx_tibsearch_search%5Baction%5D=download&tx_tibsearch_search%5Bcontroller%5D=Download&tx_tibsearch_search%5Bdocid%5D=TIBKAT%3A717891933&cHash=74e07aebe8553baac3ac1f2580585d68#download-mark.

[33] Majernik J., Gaspar S., Kmec J., Karkova M., Mascenik J. (2020). Possibility of Utilization of Gate Geometry to Modify the Mechanical and Structural Properties of Castings on the Al-Si Basis. Materials.

[34] nTopology’s Implicit Modeling Technology. https://ntopology.com/resources/whitepaper-implicit-modeling-technology/.

