# Supplementary material for: Combining Structural Optimization and Process Assurance in Implicit Modelling for Casting Parts
Source: Materials (Basel). 2021 Jul 2;14(13):3715. doi: 10.3390/ma14133715 (PMC8269844; doi:10.3390/ma14133715)
Supplement: Supplementary file 1 [file materials-14-03715-s001.zip › materials-1274515-supplementary.pdf]

materials

MDPI

Table S1. Material parameters used in the process simulation. The material properties are derived by the Flow-3D database.

Material	Parameter	Value
A380 (AlSi9Cu3)	Density (Liquid)	2460 $\frac{kg}{m^3}$
	Specific heat (Liquid)	1245.3 $\frac{J}{kg \cdot K}$
	Thermal conductivity (Liquid)	104 $\frac{W}{m \cdot K}$
	Fluid viscosity	0.0012 $\frac{kg}{m \cdot s}$
	Solidus temperature	497.3 °C
	Liquidus temperature	574.4 °C

Table S2. Material parameters used in the LPDC simulation derived by the Flow-3D database.

Material (Source: Flow3D)	Parameter	Value
A356 (AlSi7Mg0.3)	Density (Liquid)	2437 $\frac{kg}{m^3}$
	Specific heat (Liquid)	1074 $\frac{J}{kg \cdot K}$
	Thermal conductivity (Liquid)	81.4 – 197.2 $\frac{W}{m \cdot K}$
	Fluid viscosity	0.0019 $\frac{kg}{m \cdot s}$
	Solidus temperature	552.4 °C
	Liquidus temperature	608 °C
	Latent heat	$4.322 \cdot 10^5$ $\frac{J}{kg}$
H-13	Density x specific heat	$3.561 \cdot 10^6$ $\frac{kg}{m \cdot s^2 \cdot K}$
	Thermal conductivity	28.6 $\frac{W}{m \cdot K}$
	Temperature	350 °C
Ceramic	Density x specific heat	$1.800 \cdot 10^6$ $\frac{kg}{m \cdot s^2 \cdot K}$
	Thermal conductivity	1.09 $\frac{W}{m \cdot K}$
	Temperature	700 °C

Table S3. Heat transfer coefficients for the LPDC process simulation.

Material 1	Material 2	Heat Transfer Coefficient
Liquid metal	Void region	30 $\frac{W}{m^2 \cdot K}$
Liquid metal	Mold	1485 $\frac{W}{m^2 \cdot K}$
Liquid metal	Ceramic tube	2 $\frac{W}{m^2 \cdot K}$
Solidified metal	Void region	30 $\frac{W}{m^2 \cdot K}$
Solidified metal	Mold	3000 $\frac{W}{m^2 \cdot K}$

Solidified metal	Ceramic tube	1 $\frac{W}{m^2 \cdot K}$
Mold	Void region	30 $\frac{W}{m^2 \cdot K}$
Mold	Ceramic tube	5 $\frac{W}{m^2 \cdot K}$
Ceramic tube	Void region	30 $\frac{W}{m^2 \cdot K}$
